# Histopathological Evaluation of the Exposure by Cyanobacteria Cultive Containing [d-Leu^1^]Microcystin-LR on *Lithobates catesbeianus* Tadpoles

**DOI:** 10.3390/toxins10080318

**Published:** 2018-08-06

**Authors:** Osmindo Rodrigues Pires Júnior, Natiela Beatriz de Oliveira, Renan J. Bosque, Maria Fernanda Nice Ferreira, Veronica Morais Aurélio da Silva, Ana Carolina Martins Magalhães, Carlos José Correia de Santana, Mariana de Souza Castro

**Affiliations:** 1 Toxinology Laboratory, Depto. Physiological Sciences, Institute of Biology, University of Brasilia, Brasilia 70910-900, Brazil; natiela@gmail.com (N.B.d.O.); veronicasilva@gmail.com (V.M.A.d.S.); bioana.11@gmail.com (A.C.M.M.); carlosjcsantana@gmail.com (C.J.C.d.S.); mscastro69@gmail.com (M.d.S.C.); 2 Depto. Genetics and Morphology, Institute of Biology, University of Brasilia, Brasilia 70910-900, Brazil; rjbosque@go.olemiss.edu (R.J.B.); mfnf@unb.br (M.F.N.F.)

**Keywords:** [d-Leu^1^]Microcystin-LR, *Lithobates catesbeianus*, tadpoles, exposure, Histopathological evaluation

## Abstract

This study evaluated the effects of [d-Leu^1^]Microcystin-LR variant by the exposure of *Lithobates catesbeianus* tadpole to unialgal culture *Microcystis aeruginosa* NPLJ-4 strain. The Tadpole was placed in aquariums and exposed to *Microcystis aeruginosa* culture or disrupted cells. For 16 days, 5 individuals were removed every 2 days, and tissue samples of liver, skeletal muscle, and intestinal tract were collected for histopathology and bioaccumulation analyses. After exposure, those surviving tadpoles were placed in clean water for 15 days to evaluate their recovery. A control without algae and toxins was maintained in the same conditions and exhibited normal histology and no tissue damage. In exposed tadpoles, samples were characterized by serious damages that similarly affected the different organs, such as loss of adhesion between cells, nucleus fragmentation, necrosis, and hemorrhage. Samples showed signs of recovery but severe damages were still observed. Neither HPLC-PDA nor mass spectrometry analysis showed any evidence of free Microcystins bioaccumulation.

## 1. Introduction

Cyanobacterial blooms have been reported to occur in both natural and artificial water bodies and have caused severe problems for wildlife and livestock as well as humans [1,2]. Some factors contributing to a bloom formation include increased concentrations of biologically available forms of nitrogen and phosphorous in the water source, as well as high temperatures and pH, and calm weather conditions [3]. 

Due to the toxicity problems associated with numerous cyanobacterial species, a significant number of scientific papers mainly describe the occurrence and the toxins of toxic blooms, bioaccumulation and toxicity focusing on aquatic invertebrates, particularly mollusks and fishes. There are almost no reports of the effects of cyanotoxins on amphibians. The fact that the most amphibian tadpole species develop in waters, and the skin is the main osmoregulator organ and intimately connected with the aquatic environment, make them vulnerable to toxic action of some cyanobacteria blooms.

*Rhinella* (*Bufo*) *marina* tadpoles exposure for 7 days to *Cylindrospermopsis raciborskii* live culture containing cylindrospermopsin (232 µg/L) had 66% of mortality. In despite, no tadpole mortality were observed in 14 days cylindrospermopsin toxin (400 µg/L). Longer exposure to higher cylindrospermopsin concentration of *C. raciborskii* live culture resulted in an accumulation of over (400 µg/g ww) by the tadpoles [4]. In *Ambystoma mexicanum*, *Triturus vulgaris*, *Rana ridibunda* no effects were recorded during embryonic development following exposure MC-LR, -YR and saxitoxin. Although the crude extracts of cyanobacteria induced craniofacial malformations and gills hyperplasia leading to embryos death [5]. *Xenopus laevis* tadpoles fed with lyophilized cyanobacterial biomass containing Microcystin-LR (MC-LR) were unable to bioaccumulation of MC-LR, and no significantly affected in the development was observed [6]. Although Dvořáková et al. [7] demostrated in a 96 h *Xenopus laevis* embryos teratogenesis assay that MC-LR caused weak lethality but the cyanobacterial biomass containing MC-LR caused significant embryos lethality.

The fact is that there is a lack of information on amphibian biology when they are exposed to natural cyanobacteria blooms, cyanobacteria unialgal cultives or isolated cyanotoxins.

## 2. Results

### 2.1. HPLC-PDA and MALDI-TOF Analysis 

**Figure 1:**
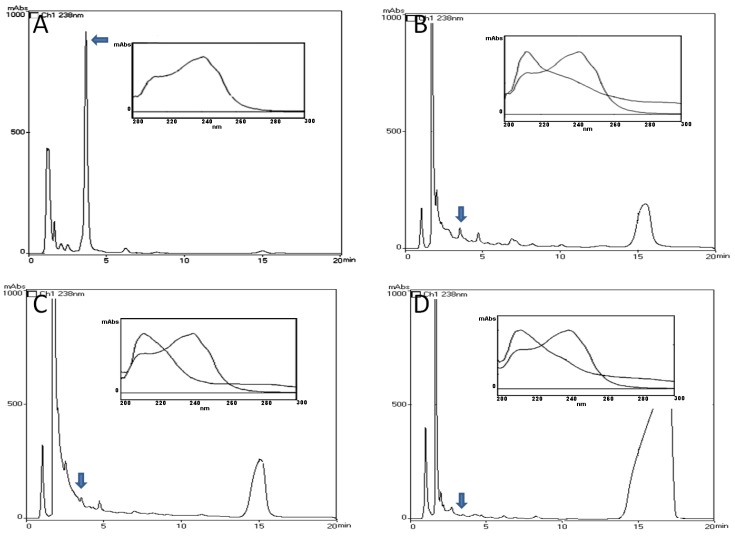
Chromatogram profile (238 nm) in HPLC-PDA system of (**A**) *Microcystis aeruginosa* NPLJ-4 culture, the arrow indicates [d-Leu^1^]Microcystin-LR at 4.5 min of retention time (Ferreira et al., 2010), the insert is MC spectra in range of 200–300 nm, (**B**) Liver, (**C**) Intestinal tract and (**D**) Muscle extracts of *L. catesbeianus* tadpole of 16 days exposed to *Microcystis aeruginosa* NPLJ-4 cells, the arrow indicates the retention time of 4.5 min, the insert is the comparison of MC spectra with fraction in 4.5 min of retention time.

In HPLC-PDA analysis of Liver, Intestinal tract and Muscle extracts of *L. catesbeianus* tadpole exposed to *M. aeruginosa* cells (Disrupted or not), no chromatographic fraction with same retention time or UV spectra (200–300 nm) of any five Microcytins (MCs) as described in Ferreira et al. [8] for *Microcystis aeruginosa* NPLJ-4 were evidenced (Figure 1). In MALDI-TOF analysis no mass components referring to any MCs described in Ferreira et al. [8] for *Microcystis aeruginosa* NPLJ-4 were found. Due to the covalent linkage between MCs and protein phosphatase, these both methods only detect free MCs available into the tissues, and not the covalently bound toxin [9], so we suggested that *Lithobates catesbeianus* tadpoles cannot accumulate microcystins as free toxins.

### 2.2. Histology Analysis

#### 2.2.1. Liver Histology

##### Control Group

**Figure 2:**
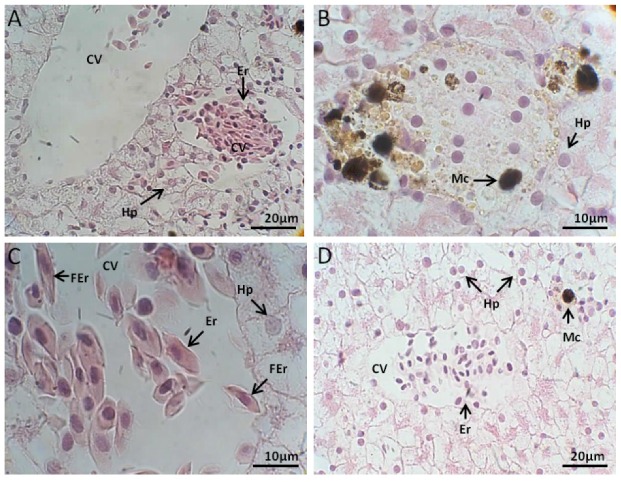
(**A**) *L. catesbeianus* tadpole liver control showing central lobe vein, erythrocytes and leukocytes, hepatocytes with uniform and well defined nuclei (40×). (**B**) giant macrophage performing phagocytosis (100×) in hepatic animal tissue 16 days exposed to *M. aeruginosa* cells. (**C**) Centrilobular vein view with morphologically altered erythrocyte micronuclei (100×), tadpoles 16 days exposed to *M. aeruginosa* disrupted cells. (**D**) Centrilobular vein with wrinkled appearance, hepatocytes with regular nuclei (40×), after a recovery period of 15 days without contact with the cyanobacteria extract. (CV) Central Vein; (Er) erythrocyte; (Hp) Hepatocytes; (Mc) macrophage, (FEr) Falciform erythrocyte.

The microscopic analysis indicated that *L. catesbeianus* tadpole liver is covered by mesothelium underlaid by a conjunctive tissue thin layer, the hepatic serosa, that coats the gland externally with no evidenced of the parenchyma division into well-defined lobules. Reticulum staining revealed that the parenchyma was supported by delicate reticular fibers surrounding hepatic cells plates intercalated by the sinusoids capillaries that converge to a central vein endowed with an endothelium. The hepatocytes was polyhedral in shape and their sizes vary. Nuclei were observed in the cells central region, but some of them were shifted toward the edge. The cytoplasm containing granules viewed as small vacuoles appeared little eosinophilic when analyzed by the H&E staining technique. The presence of granulocytes and single melano-macrophages, components of the reticulo-histiocytic system of the liver localized predominately in the sinusoid space, were observed in all of the individuals. Melano-macrophages are cells with diverse functions, including the synthesis of melanin, phagocytosis and free radicals neutralization and found numerous in amphibians. The interstices portal tracts were supported by abundant conjunctive tissue. Most of the tracts contain a bile duct, at least one vein branch and many arteries (Figure 2A).

##### Exposed to *M. aeruginosa* Cells

Macroscopically, liver showed hypertrophy with increasing exposure time to both treatments: culture of cyanobacteria and toxins after cells lyses. Changes in color and texture of the liver were also noted characterized by variation in tone dark red and firm texture going to ocher tones and looks brittle. The histological analysis showed hepatocytes highly vacuolated and at the fourth day their nucleoli were fragmented, showing characteristics of apoptosis (Figure 2B). Microcystin caused the loss of normal hepatocyte structure suggesting advanced necrosis stage, observed an eosinophilic retracted cytoplasm and the nucleus was fragmentation whereby its chromatin is distributed irregularly throughout the cytoplasm indicating karyorrhexis or karyolysis. The necrosed hepatocytes were invaded by numerous neutrophil granulocytes which were infiltrated in the parenchyma. It was also displayed a greater number of melano-macrophage filled with phagocytosed cell debris. Hepatic sinusoids presented a narrow lumen and an enlargement of the interspaces was also observed. The central veins were enlarged and fibrosed and their endothelium become progressively degenerate, and signs of hemorrhage began to appear. The portal structures formed of terminal portal veins, arterioles and biliary ducts were slightly fibrosed. 

Intense perisinusoidal fibrosis was observed around the granulomatous areas. As a consequence of hepatocytes necrosis, and active hepatic regeneration, the common hepatic lobes shape changed, appearing more rounded and shortened, comparatively to the controls. The Kupffer cells appeared a yellow-golden granular pigment, suggesting lipofuscin accumulation within the cytoplasm. Lipofuscin is pigment granules composed of lipid-containing residues of lysosomal digestion, frequently observed at the biliary pole of the hepatocytes. The lesions worsened after a longer exposure and apoptotic cells increased in number.

##### Exposed to *M. aeruginosa* Disrupted Cells

Exposure to the cells extract of *M. aeruginosa* caused injuries often similar to those observed when exposed to intact cells. Already the three-day exhibition revealed the first cyanotoxins effects, such as sinusoids dilatation near the central vein, gaps in the vein endothelium, hepatocytes with nucleus displaced, sometimes joined the cytoplasmic membrane, and increasing of vacuoles in the hepatocytes cytoplasm. At nine days of exposure occurred the disruption in the central veins endothelium. Melano-macrophages increased in size and become polynuclear cells. The misshapen appearance of sinusoids culminated in congestion and hemorrhage. Bile ducts appeared lacerated with granulocytes in the periphery (Figure 2C) revealed the most significant liver effects caused by the action of cyanotoxin after 12 days of exposure. Between 12 and 16 days the lesions get worse with more melano-macrophages polynuclear cells and increased number of granulocytes in the connective tissue surrounding bile ducts and large vessel size. The periphery of the liver was necrotic.

##### Recovery

At the end of recovery time, the remaining individuals showed the volume approached the size of the control animals and reddish. There was an improvement on the periphery of the body structure when compared to that observed at the end of exposure, despite the presence of vacuolated hepatocytes and necrotic lesions. A large number of melano-macrophages cells were still present leading to the lysis of the cells. After the recovery period of ten days, staining was most evident and connective tissue and muscle reconstitute the blood vessel walls (Figure 2D).

#### 2.2.2. Intestinal Tract Histology

##### Control Group

**Figure 3:**
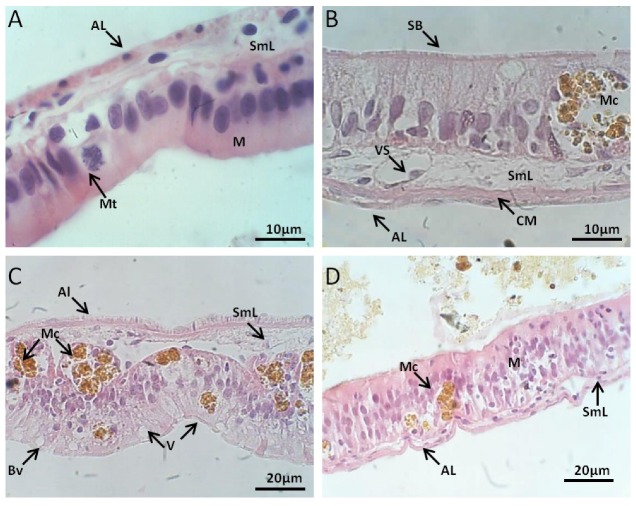
(**A**) *L. catesbeianus* tadpole Intestinal Tract control showing the structure of the layers and the uniformity of the cells (100×). (**B**) View of the striated border of the organ absorptive cells exposed for 16 days to *M. aeruginosa* cells (100×). (**C**) Loss of cell adhesion and macrophages performing phagocytosis after 16 of exposure to *M. aeruginosa* disrupted cells (40×). (**D**) Organ layers with better structured absorptive cells after recovery period of 15 days without contact with the cyanobacteria extract (40×). (AL) adventitious layer, (SmL) submucosal layer, (M) mucosa, (Mt) mitosis, (SB) striatum border, (BV) blood vessel, (ML) muscular layer, (Mc) macrophage, (Vc) vacuole, (V) villus.

The simple columnar epithelium retained a brush border and apoptotic cells were seldom found. The enterocytes showed no morphological change and goblet cells were abundant. Loose connective tissue was thin and showing fibroblast-like cells. The muscular layer was quite thin. The serous layer composed of simple squamous epithelium was difficult to visualize on sections stained with HE (Figure 3A).

##### Exposed to *M. aeruginosa* Cells

The first analysis showed granulocytes increased number through the migration of cells from the deeper layers of tissue to the base of the epithelial tissue. The enterocytes showed lesions as the presence of cytoplasmic vacuoles. Also noticeable was the increased fibrosis in the connective tissue layer beneath the epithelium which appeared thicker. An intense blood supply was observed around the granulomatous areas. On subsequent days of exposure, the intestine showed signs of change as a greater number of apoptotic epithelial cells.

##### Exposed to *M. aeruginosa* Disrupted Cells

After five days of exposure to extract the absorptive cells and goblet cells that form the simple cylindrical epithelium apparently remain with integrity and preserved (Figure 3B). The enterocytes were attached to basal lamina and despite the abundant vascularization, connective tissue showed signs of fibrosis. No changes were observed in the muscle layer and serosa. The main changes brought about by the action of the extract of *M. aeruginosa* in the intestine occurred after six days of exposure. At that point, clusters of melano-macrophage were evident among the epithelial cells. These cells had an elongated aspect, vacuoles in apical part and loss of adhesion. It was observed an increase in melano-macrophages throughout the intestinal wall. After 16 days of exposure, these aspects were exacerbated by the loss of demarcation between the epithelial cells, culminating in degeneration process with nuclei displaced adhered to the cytoplasmic membrane and necrosis (Figure 3C).

##### Recovery

After the period of recovering, the number and size of melano-macrophages decrease. The cells adhesion and delimitation were still limited. The blood vessels remained narrow and the intestine wall appeared thin (Figure 3D). 

#### 2.2.3. Muscle Histology

##### Control Group

**Figure 4:**
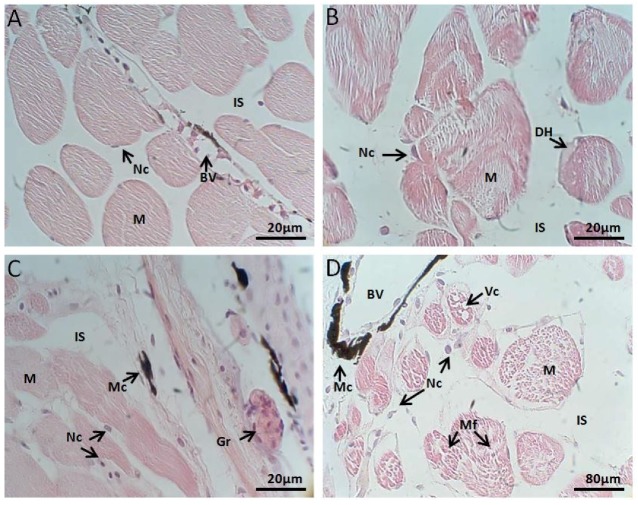
(**A**) *L. catesbeianus* tadpole muscle control showing structure and organization of muscle tissue (40×). (**B**) Animal tissue exposed for 16 days to *M. aeruginosa* cells (40×). (**C**) Longitudinal section of animal tissue exposed for 16 days to expose to *M. aeruginosa* disrupted cells (40×). (**D**) Transverse section of recovering animal tissue after 15 days without contact with the extract (10×). (M) myocyte, (IS) intercellular space, (Nc) nucleus, (BV) blood vessel, (HD) degradation hyaline, (Mc) macrophage, (Gr) granulocyte, (Vc) vacuole, (Mf) myofibril.

This tissue had transverse striations, multinucleated cells, peripheral nuclei and other characteristics of skeletal muscles. The muscle fibers or myocytes showed a surrounding connective tissue (Figure 4A).

##### Exposed to *M. aeruginosa* Cells

In muscle fibers, after two days of exposure, staining becomes weaker, and myofibrils, the smallest contractile units, were poorly visible with the light microscope. The lesions were more pronounced with exposure time, increasing the intercellular spaces between the muscle fibers and cytoplasmic derangement with no evident cross-striation (Figure 4B). At the end of the toxicity experiment, muscle fibers had signs of fragmentation and cytoplasmic lysis with cells contents release.

##### Exposed to *M. aeruginosa* Disrupted Cells

The weak staining also allows us to observe a loss of striations which implies the loss myofibril arrangement. At half time exposure tissue abnormalities were more advanced, when necrosis and the intercellular spaces increased. Due the loss of intracellular adhesion, muscle layers cells were separated from each other. Signs of nuclear fragmentation and necrosis were observed (Figure 4C).

##### Recovery

Although we observed an improvement in the stain that has become less pale, tissue recovery, after fragmentation of muscle fibers, was insignificant (Figure 4D). 

## 3. Discussion

There are numerous studies describing the harmful effects of Microcystins on adult fish [10,11], as well as embryonic and post-hatch development [12]. In *Cyprinus carpio*, histopathological changes were observed in the liver and gastrointestinal tract and increased in severity with post-dose time [11]. The tissues of Silver carp *Hypophthalmichthys molitrix* exposed *Microcystis aeruginosa* NPLJ4 were characterized by significant damages such as cells dissociation, necrosis and hemorrhage [8]. In spite of numerous studies with aquatic animals, there is a lack of work relating the toxic effects of cyanotoxins on amphibians. According to our observation, the exposal to a *Microcystis aeruginosa* containing d-Leu-Microcistin-LR cultures cells (disrupted or not) promotes severe damage liver, muscle and intestinal tract of *L. catesbeianus* tadpole. The organization and morphology of organs and tissues samples were basically the same, the loss of sinusoidal integrity, plasma membrane integrity, the loss of normal cell-to-cell adhesions, loss of cell shape, necrosis, apoptosis and cytoplasmic vacuolization. 

MCs interact with protein phosphatases at a molecular level by forming a covalent linkage between Mdha residue and the phosphatase’s cysteine residue [13,14,15,16,17,18]. Perhaps all these effects are being centralized in the destabilization of the cytoskeleton as a direct action of cyanotoxin inhibition of protein phosphatases [19,20]. 

In addition, recent studies have revealed that the exposure hepatocytes to MCs induce hepatocytes cytoskeleton components rearrangement or collapse, mainly microtubules, micro- and intermediate filaments [20,21,22,23,24,25,26], and similar effects have also been observed in others tissues [27,28,29,30,31,32,33,34]. MCs can induce cytoskeletal disruption by affects the expression of cytoskeletal and cytoskeleton associated proteins as downregulated de expression of actin and tubulin, and hyperphosphorylation several microfilament-associated proteins, like Vimentin, Ezrin and VASP [27,33]. 

In contrast to the results of the exposure of *Rhinella (Bufo) marina* tadpoles to live culture containing Cylindrospermosin that caused 66% mortality [4], the treatment (disrupted cells or not) caused no mortality of *L. catesbeianus* tadpoles during the exposure period and subsequent detoxification. Although, the damage level was characterized by a time-dependent exposure. 

*Rhinella* (*Bufo*) *marina* also accumulated cylindrospermopsin only when exposed to *C. raciborskii* culture, but not when exposed to the culture lysed cells. In this work the authors argue that the difference in results is related to the way the toxin is or not absorb. The *C. raciborskii* live cells culture was eaten by tadpole and the absorption was intestinal tract, while the *C. raciborskii* lysed cells the cylindrospermopsin was not absorbed by the skin. 

Even though the methodology employed in this work is limited to determination of unbound MC concentration, interestingly, *L. catesbeianus* tadpoles do not appear to accumulate Microcystins in its unbounded form in tissues since we could not identify the presence of any Microcystins in either treatment, disrupted cells or not. In a similar way, *Xenopus laevis* tadpoles fed with cyanobacterial biomass containing MC-LR did not bioaccumuled this toxin [6].

Amphibian tadpoles appear to be very susceptible to the cytotoxic effects of Microcystins, but also appear to have a high capacity for tissue regeneration after acute intoxication. To our knowledge, this is the first report of cytotoxic effects on amphibian tadpoles, with histopathological description of the effects of acute intoxication by a Microcystin variant, as well as the concomitant evaluation of the possibility of bioaccumulation

## 4. Materials and Methods 

### 4.1. Cyanobacteria Culture and the Experimental Animals

Unialgal *M. aeruginosa* NPLJ4 culture were carried out in the Laboratory of Toxinology, University of Brasilia. 150 *Lithobates catesbeianus* tadpoles about 24–25 stage development according to Gosner [34], were donated by a toad breeding company nearby Brasilia-DF. Animals were distributed in 3 aquariums (84 L of each, 30 cm × 60 cm × 50 cm) with filtrated and dechlorined water, acclimatized for 20 days with 12/12 h light/darkness photoperiod, continuous oxygenation with submerged pumps, and temperature at 25 ± 2 °C. The tadpoles were fed with specific tadpole food, also kindly given by the toad breeding company.

### 4.2. M. aeruginosa NPLJ4 Toxins Characterization 

The purification and characterization protocols used were the same of Ferreira et al., [8]. *M. aeruginosa* NPLJ4 culture extract of showed the same presence of 5 microcystins, the chromatographic fraction identified as [d-Leu^1^]MC-LR showed over 90% of all microcystins detected [8]. The [d-Leu^1^]MC-LR concentration for each assay was calculated in 11 mg for 840 mL culture medium containing colonies of *M. aeruginosa* NPLJ4 at the end of the exponential growing phase.

### 4.3. Exposure of Tadpole to M. aeruginosa Cells or Cells Extract 

At the end of the acclimatization period, animals were submitted to: Aquaria No. 1, received 840 mL of culture medium containing colonies of *M. aeruginosa* in the final exponential growth phase. The concentration of cells in aquaria was the equivalent of 10^5^ cells per mL. Aquaria No. 2, received the ultrasonicated disrupted *M. aeruginosa* cell extract from 840 mL culture medium containing colonies of *M. aeruginosa* in the final exponential growth phase. As microcystins are endotoxins, the lysis by ultrasonicated disruption had intended the release of all microcystins in the growth medium. The final concentration of [d-Leu^1^]MC-LR itself were estimated in 0.13 mg/L.

During the initial period of 16 days, 5 (five) individuals, from each aquaria, were removed at 2-day intervals and euthanized by over anesthesia using a lidocaine 5% ointment applied in ventral skin, All procedures were in accordance with the Local Ethics Committee of Brasilia University (Process Number CEUA 56344/2005, project name: Microcystin Bioaccumulation in aquatic animals). The five individuals were divided into two groups, the first group consisting of three (3) individuals intended for quantification of MCs bioaccumulated by HPLC-PDA and MALDI-TOF analysis, and the second group by two (2) individuals reserved for histopathological analysis.

During the experiment the aquariums were kept continuously aerated; temperature was maintained at 25 ± 1 °C, pH ~7.0 with 12/12 h light/darkness photoperiod. After 2 days, the water was changed and the same culture volume of *M. aeruginosa* culture or cell disrupted extract was added, aiming to maintain MCs concentration in contact with animals.

After sixteen days of exposure, the remaining animals were removed to another 2 free cell aquaria and after fifteen days 5 (five) individuals were collected and euthanized following the same previously mentioned methodology to evaluate the detoxification and tissue regeneration. Animals that were not exposed to cell culture formed the control group, and were collected under the same conditions.

### 4.4. HPLC-PDA Analysis 

To the determination of unbound MCs concentration tissue samples (~5 g for liver and intestinal tract, 10 g for muscle), were extracted three times in methanol (5 mL/g). The extracts were filtered (glass fiber membrane 1.2 µm porosity), vacuum-dried, and resuspended in deionized water (5 mL). The extract cleanup was performed with solid phase extraction cartridges (Strata C18, 5 mg; Phenomenex, Torrance, CA, USA) [8]. The resultant extract was vacuum-dried and resuspended in deionized water (2 mL). The sample was filtered through a 0.22 µm polyethylene filter (GV Millex; Millipore Corporation, Billerica, MA, USA), and analyzed by a Shimadzu LC-10A HPLC system (Shimadzu, Kyoto, Japan) equipped with a SPD M10A photodiode array detector. Conditions Synergi column (4 µm Fusion-RP80, 150 × 4.60 mm, Phenomenex, Torrance, CA, USA), mobile phase 20 mM ammonium formate in 30% acetonitrile (pH 5.0), run isocratically, flowrate 1 mL/min for 30 min, UV detection at 238 nm. Toxins identification was performed by chromatograms comparison of standard of MC-LR (Sigma-Aldrich Corporation, Saint Louis, MO, USA) and [d-Leu^1^]MC-LR purificated in our laboratory, observing the following aspects: the retention time and similarity of UV spectra (200–300 nm). Concentrations of [d-Leu^1^]MC-LR, were calculated from the calibration curve (*R*^2^ = 0.9941), with detection limit of 10 ng. For bioaccumulation positive control, 1 mg of [d-Leu^1^]MC-LR was added and homogenized to untreated animal tissues, our recovery was approximately 60% (Data not shown).

### 4.5. Determination of Unbound MCs Concentration in Tissues—MALDITOF Analysis

To examine the presence of unbound MCs, the tissue samples were also submitted to Ultraflex II™ TOF/TOF (Bruker, Bremen, Germany). Samples aliquots were dissolved in TFA 0.1% and mixed with a saturated matrix solution of α-cyano-4-hydroxycinnamic acid (1:3, *v*/*v*) and directly applied onto a target (AnchorChip™, Bruker Daltonics, Billerica, MA, USA). The mass spectrometry was operating in positive reflector mode for MALDI-TOF, or LIFT mode for MALDI-TOF/TOF. Calibration was performed externally with ions of angiotensin I, angiotensin II, substance P, bombesin, insulin b-chain and adrenocorticotropic hormones (clip 1–17 and clip 18–39). Each spectrum was produced by accumulating data from 200 consecutive laser shots. 

### 4.6. Histology Analysis

Histological material for the evaluation of the cyanotoxin effects on tissues and organs morphology was fixed in 10% neutral buffered formalin (pH 7.0) for 24 h, dehydrated, in xylene and paraffin-embedded routinely. Sections of 3–5 µm thick were deparaffinized in xylene, rehydrated and stained with Hematoxylin & Eosin (H&E). Detection of histopathological changes were achieved by evaluation of general architecture of organs, cellular morphology and blood vessel histology in tissue sections viewed using optical microscope. The system for capturing images consisted of camera CCD-Iris and capture plate PixelView Station, Image Pro Express 4.0, Media Cybernetics, Scopephoto Version x86, 3.1.1.615 (ScopeTek, Goleta, CA, USA).
